# Mitochondrial Dysfunction in Lysosomal Storage Disorders

**DOI:** 10.3390/diseases4040031

**Published:** 2016-10-11

**Authors:** Mario de la Mata, David Cotán, Marina Villanueva-Paz, Isabel de Lavera, Mónica Álvarez-Córdoba, Raquel Luzón-Hidalgo, Juan M. Suárez-Rivero, Gustavo Tiscornia, Manuel Oropesa-Ávila

**Affiliations:** 1 Centro Andaluz de Biología del Desarrollo (CABD-CSIC-Universidad Pablo de Olavide), Sevilla 41013, Spain; david.cotan@hotmail.com (D.C.); marvp75@gmail.com (M.V.-P.); isadelavera@gmail.com (I.d.L.); monikalvarez11@hotmail.com (M.Á.-C.); raqueluzon@gmail.com (R.L.-H.); juasuariv@gmail.com (J.M.S.-R.); manueloropesa@hotmail.com (M.O.-Á.); 2 Department of Biomedical Sciences and Medicine, University of Algarve, Faro 8005-139, Portugal; gtiscornia@ualg.pt

**Keywords:** lysosomal storage disorders, mitochondrial dysfunction, Gaucher disease

## Abstract

Lysosomal storage diseases (LSDs) describe a heterogeneous group of rare inherited metabolic disorders that result from the absence or loss of function of lysosomal hydrolases or transporters, resulting in the progressive accumulation of undigested material in lysosomes. The accumulation of substances affects the function of lysosomes and other organelles, resulting in secondary alterations such as impairment of autophagy, mitochondrial dysfunction, inflammation and apoptosis. LSDs frequently involve the central nervous system (CNS), where neuronal dysfunction or loss results in progressive neurodegeneration and premature death. Many LSDs exhibit signs of mitochondrial dysfunction, which include mitochondrial morphological changes, decreased mitochondrial membrane potential (ΔΨm), diminished ATP production and increased generation of reactive oxygen species (ROS). Furthermore, reduced autophagic flux may lead to the persistence of dysfunctional mitochondria. Gaucher disease (GD), the LSD with the highest prevalence, is caused by mutations in the GBA1 gene that results in defective and insufficient activity of the enzyme β-glucocerebrosidase (GCase). Decreased catalytic activity and/or instability of GCase leads to accumulation of glucosylceramide (GlcCer) and glucosylsphingosine (GlcSph) in the lysosomes of macrophage cells and visceral organs. Mitochondrial dysfunction has been reported to occur in numerous cellular and mouse models of GD. The aim of this manuscript is to review the current knowledge and implications of mitochondrial dysfunction in LSDs.

## 1. Introduction

Mitochondria are double-membrane organelles that are found in most eukaryotic cells and that execute many metabolic functions including ATP synthesis through oxidative phosphorylation (OXPHOS) [1]. Mitochondria are also involved in synthesis of biomolecules, maintenance of calcium homeostasis, production of reactive oxygen species (ROS), and apoptosis activation [2]. Mitochondria are structurally complex and highly dynamic motile organelles. Mitochondria undergo constant morphological changes by the process of continuous cycles of fusion and fission that determines their morphology and most mitochondrial functions [3].

Given their central role in cellular homeostasis, mitochondrial dysfunction has been linked to many age-related disorders including mitochondrial diseases, cancers, metabolic diseases and diabetes, inflammatory conditions, neuropathy, and neurodegenerative diseases such as Alzheimer’s, Parkinson’s, and Huntington’s disease [4,5]. Currently, the hypothesis is beginning to emerge which suggests that neurodegenerative disorders share similarities in terms of underlying pathogenic mechanisms with lysosomal storage diseases (LSDs) [6]. Thus, dysfunctional mitochondria, impaired autophagy and accumulation of cytoplasmic protein aggregates are frequent alterations shared by LSDs and more common neurodegenerative disorders [7,8,9].

LSDs are a group consisting of over 50 disorders, characterized by the progressive accumulation of undigested macromolecules in lysosomes. The massive accumulation of substances affects the function of lysosomes and impairs autophagic flux which may affect the cellular quality control of organelles such as mitochondria [10].

Lysosomes are the primary disposal and recycling center of cells. Thus, lysosomes are involved in the degradation and recycling of extracellular material (via endocytosis) and intracellular material (via autophagy) [11,12].

Autophagy is an essential cellular system that consists of the degradation of cytoplasmic components within lysosomes. Autophagy pathways represent a major defense mechanism that, in addition to their role in providing cells with nutrients, also allow cell survival in response to multiple stressors.

Three general types of autophagy have been described—macroautophagy, microautophagy, and chaperone-mediated autophagy—which differ in their regulation and selectivity [13]. Macroautophagy is the process involved in the degradation of cytoplasmic organelles and cytosolic components. The best characterized kind of autophagy, macroautophagy, starts with the formation of an isolation membrane enveloping cytoplasmic cargoes to generate an autophagosome. The autophagosome then fuses with the lysosome to form an autophagolysosome. The degradation takes place in the autophagolysosome by hydrolytic enzymes. Abnormal lysosomal function in LSDs has been associated with alterations in macroautophagy due to impaired autophagosome-lysosome fusion or deficient degradation of autophagy substrates [14].

Lysosomes are essential for autophagy, and autophagic clearance of dysfunctional mitochondria represents an important element of mitochondrial quality control [15]. It is easy to speculate that lysosomal dysfunction such as in LSDs [16] leads to the abnormal accumulation of non-hydrolysed autophagy cargos such as mitochondria and other organelles. Indeed, accumulation of abnormal and dysfunctional mitochondria has been reported in various types of LSDs [8].

Once the mitochondrial damage becomes too extreme and causes dissipation of mitochondrial membrane potential (ΔΨm), damaged parts of mitochondria cannot fuse with the adjacent healthy mitochondria [17]. The segregation of dysfunctional mitochondria from the rest of the mitochondrial network limits the damage and allows selective elimination by mitophagy [18]. Autophagic flux is a dynamic process that involves autophagosomes formation, their fusion to lysosomes and the digestion of the autophagic cargo [19]. Importantly, impaired autophagic flux will also affect mitophagy and perturb the clearance of dysfunctional mitochondria [20].

In LSDs, both lysosomal dysfunction and reduction of autophagic flux have a major impact on mitochondrial function. The dysregulation of autophagic flux may reside at the stage of autophagosome clearance, the final step in the autophagy process in which the lysosome plays a pivotal role. A significant number of damaged mitochondria and autophagosomes remained in the cytosol, suggesting inefficient and incomplete autophagic flux.

Alternatively, lysosomal enzyme deficiency may translate into mitochondrial dysfunction by cellular lipid trafficking alterations [21,22]. In healthy cells, membrane lipid components are hydrolyzed inside lysosomes. In LSDs, this process is disturbed leading to lysosomal trapping of lipid compounds and liberation of mal-processed lipids. Trapping of substrates and their metabolites in the lysosome may lead to an altered content of the recycled lipids, which has a direct effect on the lipid composition of membranes in different organelles, for example, the inner mitochondrial membrane. As respiratory chain complexes I, II, III, and IV are embedded in the inner mitochondrial membrane, an altered lipidic composition of mitochondrial membranes would, therefore, result in mitochondrial dysfunction [8].

Herein, we will review the current information regarding mitochondrial dysfunction in several LSDs, with particular interest in data derived from various cellular and animal models in Gaucher disease (GD).

## 2. LSDs Associated with Nonmembrane-Bound Lysosomal Hydrolases

### 2.1. Glycogenosis Type II (Pompe Disease)

Pompe disease—a lysosomal glycogen storage disease caused by deficiency of acid alpha-glucosidase—is characterized by glycogen accumulation in muscle and peripheral nerves. Recently, the contribution of mitochondrial dysfunction in the pathophysiology of Pompe disease has been recognized [23]. Multiple mitochondrial defects such as mitochondrial calcium overload, increase ROS production, mitochondrial depolarization, as well as a decreased oxygen consumption and ATP production have been demonstrated in muscle cells of mouse and human models of Pompe disease [24]. Furthermore, dysfunctional mitochondria with swollen cristae have been recently observed in induced pluripotent stem cells (iPSCs) derived from fibroblasts of patients with Pompe disease [25]. Mitochondria have also been visualized in autophagic vacuoles in muscle biopsies of adult Pompe patients [26]. The aberrant mitochondria found in Pompe patients were sequestered in autophagic vesicles that were unable to reach the lysosomes. While suppression of autophagy in skeletal muscle in wild-type mice leads to accumulation of dysmorphic mitochondria [27], it is still unclear whether mitochondrial abnormalities occur regardless or because of autophagy blockage.

### 2.2. Multiple Sulphatase Deficiency (MSD)

MSD, is a rare LSD caused by mutations in the SUMF1 gene, resulting in an accumulation of sulphatides, sulphated glycosaminoglycans, sphingolipids and steroid sulphates. Defects in both autophagy and mitophagy have been observed in the liver and brain of murine models of MSD [14,28].

Mitochondria in these models were found to be fragmented with reduced ΔΨm and ATP production, indicating a generalized mitochondrial dysfunction.

### 2.3. Mucopolysaccharidoses (MPS)

MPS is the heterogeneous group of LSDs that result from a deficiency in lysosomal enzymes responsible for glycosaminoglycan (GAG) degradation. Mucopolysaccharidosis III type C is a severe neurologic disease caused by mutations in the gene that encodes the enzyme heparan acetyl-CoA: alpha-glucosaminide *N*-acetyltransferase (HGSNAT). It has been proposed that HGSNAT deficiency and lysosomal accumulation of heparan sulfate in microglial cells followed by their activation and cytokine release result in mitochondrial dysfunction in the neurons in an animal model of the MPS IIIC [29]. In another study, it was confirmed that in the mouse model of mucopolysaccharidosis III type C, heparan sulfate accumulation caused mitochondrial dysfunction and accumulation in autophagosomes [30,31]. Accumulation of fragmented mitochondria along with increased inflammation and cell death have been observed in fibroblasts from MPS VI patients and in tissues of MPS VI rats [32].

### 2.4. Mucolipidoses (ML) Types I–III

ML is the collective name for a group of autosomal recessive diseases characterized by accumulation of membranous lipid inclusions in patient cells. There are four types of ML. Types I–III involve the miss-targeting of lysosomal lipid hydrolases, resulting in inefficient processing of endocytosed lipids [33]. Lysosomal storage possibly causes autophagic impairment that leads to accumulation of aberrant mitochondria. In the case of ML-II [9], autophagic and mitochondrial impairment has been reported in skin fibroblasts [34], mice, and an autopsy case [35,36].

### 2.5. G(M1)-Gangliosidosis

GM1-gangliosidosis is caused by an inherited deficiency of the lysosomal enzyme GM1-β-galactosidase (beta-gal) that results in the accumulation of GM1 ganglioside. GM1-ganglioside accumulates in the glycosphingolipid-enriched microdomain (GEM) fractions of mitochondria-associated ER membranes or MAMs in the brains of mouse models of GM1-gangliosidosis. There, it has been proposed that it interacts with the phosphorylated form of IP3 receptor-1, affecting the activity of this channel and triggering a Ca^2+^-mediated-ER stress response. Consequently, calcium is then taken up by the mitochondria, leading to calcium overload and activation of the mitochondrial apoptotic pathway [37]. Mitochondria isolated from beta-gal (−/−) mouse astrocytes were morphologically abnormal and had a decreased ΔΨm [38].

### 2.6. Fabry Disease

Fabry disease is characterized by lysosomal storage of globotriaosylceramide. Accumulation of this compound results from dysfunction of α-galactosidase A encoded by the *GLA* gene located on the X chromosome. Reduced activities of respiratory chain complexes and impaired mitochondrial energy have been reported in fibroblasts from patients with Fabry disease [39].

### 2.7. Farber Disease

Farber disease is an extremely rare autosomal recessive LSD characterized by a deficiency in the enzyme ceramidase, which leads to ceramide accumulation in lysosomes. It has been suggested that the ceramide that accumulates in severe forms of Farber disease cells is sequestered to distinct membrane subdomains. These domains are located in membranes of the endomembrane system, and also in two unexpected locations, namely, the mitochondria and the plasma membrane, which may explain some of the cellular pathology observed in this severe LSD [40].

### 2.8. Gaucher Disease

GD, the most prevalent LSD, is caused by mutations in the GBA1 gene that causes defective activity of β-glucocerebrosidase (GCase). Decreased enzyme activity and/or instability of GCase provoke accumulation of glycolipids in the lysosomes of macrophages and internal organs. GD can be classified into 3 variants depending on age at onset and the presence of neurological manifestations. Gaucher patients without central nervous system (CNS) involvement are classified as type I, while those with CNS involvement are type II or type III. Furthermore, mutations in the gene GBA1 are a risk factor for Parkinson’s disease and dementia with Lewy bodies [41]. Common underlying defects of these diseases such as impaired autophagy and mitochondrial dysfunction, suggest a possible mechanistic relationship with the pathophysiology of Gaucher disease.

The loss of GCase activity causes defects in the autophagy-lysosome pathway and mitochondrial function in GD [42]. Inhibiting the enzymatic activity of the GCase enzyme with conduritol beta epoxide (CBE), or silencing the GBA1 gene in human neuroblastoma cells SH-SY5Y, caused mitochondrial dysfunction in these cell models, showing reduced activity of mitochondrial ETC and mitochondrial depolarization and fragmentation associated with increased levels of ROS [43]. The analysis of individual cells as astrocytes and neurons derived from a mouse model of Gaucher type II showed a reduction ΔΨm and activities of respiratory complex I, II and III and increased mitochondrial fragmentation [44].

Furthermore, brain histological sections from models with neuronal variants of Gaucher disease (nGD) revealed disruption of mitochondrial cristae and the presence of fragmented mitochondria with rounded morphology [45]. These animal models also showed reduced oxygen consumption and low ATP levels. In addition of GlcCer and GlcSph accumulation, protein aggregates of α-synuclein (α-Syn) and amyloid beta precursor protein were also observed in the cortex, hippocampus, stratum and substantia nigra of the nGD mice. Interestingly, protein aggregates co-localized with mitochondria, suggesting that they can directly affect mitochondrial function. Accumulation of α-Syn has been found that inhibits complex I of mitochondrial ETC, reduces ΔΨm and alters calcium homeostasis [46]. Similar results have been obtained in cultured neurons treated with synthetic α-Syn (18). Dysregulation of calcium can also have an effect on mitochondrial function, either directly causing mitochondrial depolarization or oxidative damage by mitochondrial ROS generation. Calcium levels have been found to be altered in iPSC-differentiated Gaucher neurons [47].

Therefore, the defective function of GCase and accumulation of GlcCer, GlcSph and protein aggregates are expected to be risk factors to cause mitochondrial dysfunction.

Other animal models of Gaucher disease as GBA KO fish medaka (*Oryzias latipes*), showed colocalization of mitochondria with autophagic marker LC3, suggesting the degradation of mitochondria in autophagosomes [48]. On the other hand, the GBA KO Zebrafish (*Danio rerio*) model presented mitochondrial dysfunction and reduced autophagic flux, deterioration of the activity of complexes III and IV of mitochondrial ETC, and reduced protein expression of complex I (subunit NDUFA9) and IV (Cox4i1) [49]. iPSC-differentiated Gaucher type II neurons also showed mitochondrial dysfunction, reduced autophagic flux, and accumulation of autophagosomes [47].

Mitochondrial dysfunction has also been reported in GD fibroblasts and GD mouse models. Dermal fibroblasts from three GD patients (L444P/L444P) showed reduced activity of complex I, II, III and II + III of the ETC [50]. Mitochondrial dysfunction was associated with reduced mitochondrial membrane potential, increased reactive oxygen species (ROS), mitophagy activation and impaired autophagic flux. Both abnormalities, mitochondrial dysfunction and deficient β-glucocerebrosidase activity, were partially restored by supplementation with coenzyme Q_10_ (CoQ) or a l-idonojirimycin derivative, *N*-[*N*’-(4-adamantan-1-ylcarboxamidobutyl)thiocarbamoyl]-1,6-anhydro-l-idonojirimycin (NAdBT-AIJ), and more markedly by the combination of both treatments. These data suggest that targeting both mitochondria function by CoQ and protein misfolding by pharmacological chaperones can be promising therapies in neurological forms of GD. These findings support the hypothesis that secondary mitochondrial dysfunction by GlcCer accumulation aggravates the effects of the L444P/L444P mutation and therefore, the improvement of mitochondrial function may improve the activity of the mutant GCase [50].

On the other hand, persistent GlcCer accumulation in neurons and glial activation causes a continuous chronic inflammation that may contribute to neuronal cell death [45,51]. For example, Gaucher mouse models generated by CBE treatment showed the presence of neuroinflammation, α-Syn accumulation, synaptic dysfunction and neurodegeneration [52]. Finally, excessive production of free radicals such as nitric oxide by astrocytes and activated microglia in GD can also impact on some of the mitochondrial abnormalities observed in GD models [42].

## 3. LSDs Associated with Integral Lysosomal Membrane Proteins

### 3.1. Niemann-Pick Disease

Niemann–Pick type C (NPC) disease is an LSD caused by mutations in the NPC1 or NPC2 genes, leading to the accumulation of cholesterol and glycosphingolipids in late endosomes and lysosomes [53]. It has been reported that NPC1 mutant fibroblasts showed deregulation of the function and organization of the mitochondrial network [54]. Increased mitochondrial cholesterol is potentially detrimental for mitochondrial function [55]. Recent studies indicate that while the production of autophagosomes is enhanced, progression of the autophagic process is stalled, leading to the accumulation of defective mitochondria in NPC1-deficient neurons [56,57].

Other studies have demonstrated that cholesterol content in mitochondrial membranes is significantly increased in NPC1 mouse brains and neural cells. In addition, the ΔΨm, the activity of ATP synthase, and the level of ATP are markedly decreased in NPC1 mouse brains and neurons [58]. These results suggest that mitochondrial dysfunctions and subsequent ATP deficiency, which are induced by altered cholesterol metabolism in mitochondria, may be responsible for neuronal impairment in NPC1 disease.

### 3.2. Mucolipidoses (ML) Type IV

ML type IV is a genetic lysosomal storage disease associated with degenerative processes in the brain, eye, and other tissues. ML type IV is linked to mutations in the ion channel, mucolipin 1 (MCOLN1)—a protein that plays a role in lysosomal/endosome function [59]. Significant mitochondrial fragmentation has been observed in mucolipidosis type IV [60].

### 3.3. Cystinosis

Cystinosis was the first lysosomal storage disease recognized to be due to defective lysosomal membrane transport, and it serves as a prototype for a small group of lysosomal transport disorders. The disease results in intracellular accumulation of cystine in all organs and tissues.

Further studies demonstrated a significant decrease in mitochondrial ATP generation with increase ROS in cystinotic cells [61]. Electron microscopy images showed the presence of aberrant mitochondria and impaired mitophagy with a high number of autophagic vacuoles and reduced number of mitochondria in nephropathic cystinosis [62].

## 4. LSDs Associated with Other Defects

### 4.1. Neuronal Ceroid-Lipofuscinoses

The neuronal ceroid lipofuscinoses, also known as Batten disease, are a group of LSDs that can arise from genetic mutations within 1 of 14 different genes [63]. Pathological hallmarks of NLCs include the accumulation of autofluorescent lipopigments resembling ceroid and lipofuscin.

Although the biochemical defects seem to affect primarily lysosomes, the connection between this and neuronal death is not completely understood. The pathogenic mechanisms have been studied particularly in two animal models: the English setter dog and the New Zealand South Hampshire sheep (OCL6). In these models, and some of the human entities, there is evidence of mitochondrial dysfunction [64]. This includes the accumulation of subunit c of ATP synthase as a component of storage material in at least six of eight genetic forms of the disease, and the presence of abnormal mitochondria associated with the loss of neurons in areas of the brain metabolically active. Mitochondrial dysfunction comes from functional tests in fibroblasts and, in animal models, isolated liver mitochondria. In addition, decreased oxygen consumption and mitochondrial ECT enzymes have been also reported in neurons from a mouse model of neuronal ceroid lipofuscinosis [65].

### 4.2. Implications for Therapy in LSDs

Given that defects in mitochondrial function and oxidative stress have been demonstrated to play a role in the pathogenesis of many LSDs, we envisioned that treatment with antioxidants and mitochondria energizers such as CoQ could exert beneficial therapeutic effects. The critical role of CoQ in mitochondrial bioenergetics and its well-known antioxidant properties represent the main rationale for its clinical use, although some of its effects may be related to a gene induction mechanism [66]. Interestingly, CoQ is also able to cross the blood–brain barrier in animal models [67,68], although central nervous system penetration of oral CoQ in humans has not been studied [69]. Therefore, the adjuvant treatment of CoQ in addition to enzyme replacement and/or substrate reduction therapy may represent a therapeutic strategy in LSDs.

## 5. Conclusions

The convergence of impaired autophagic flux and altered composition of cell membranes contributes to mitochondrial dysfunction in LSDs. The alteration of mitochondrial morphology and decreased respiratory chain activity varies in both extent and characteristics in tissues and isolated mitochondria among these diseases.

We propose that lysosomal dysfunction interferes with the both the function and clearance of damaged mitochondria and that these critical pathways converge in the pathogenesis in LSDs.

Finally, numerous observations in both cellular and animal models of GD suggest that mitochondrial alterations might be involved in the pathophysiology of this lysosomal disorder and may aggravate its clinical presentation.
